# Structure and vacancy distribution in copper telluride nanoparticles influence plasmonic activity in the near-infrared

**DOI:** 10.1038/ncomms14925

**Published:** 2017-03-30

**Authors:** Tom Willhammar, Kadir Sentosun, Stefanos Mourdikoudis, Bart Goris, Mert Kurttepeli, Marnik Bercx, Dirk Lamoen, Bart Partoens, Isabel Pastoriza-Santos, Jorge Pérez-Juste, Luis M. Liz-Marzán, Sara Bals, Gustaaf Van Tendeloo

**Affiliations:** 1 EMAT, University of Antwerp, Groenenborgerlaan 171, Antwerp B-2020, Belgium; 2 Department of Materials and Environmental Chemistry, Stockholm University, Stockholm SE-106 91, Sweden; 3 Departamento de Quimica Fisica, Universidade de Vigo, Vigo ES-36310, Spain; 4 CMT Group, Department of Physics, University of Antwerp, Groenenborgerlaan 171, Antwerp B-2020, Belgium; 5 Bionanoplasmonics Laboratory, CIC biomaGUNE, Paseo de Miramon 182, Donostia-San Sebastian ES-20014, Spain; 6 Ikerbasque, Basque Foundation for Science, Bilbao ES-48013, Spain

## Abstract

Copper chalcogenides find applications in different domains including photonics, photothermal therapy and photovoltaics. CuTe nanocrystals have been proposed as an alternative to noble metal particles for plasmonics. Although it is known that deviations from stoichiometry are a prerequisite for plasmonic activity in the near-infrared, an accurate description of the material and its (optical) properties is hindered by an insufficient understanding of the atomic structure and the influence of defects, especially for materials in their nanocrystalline form. We demonstrate that the structure of Cu_1.5±*x*_Te nanocrystals can be determined using electron diffraction tomography. Real-space high-resolution electron tomography directly reveals the three-dimensional distribution of vacancies in the structure. Through first-principles density functional theory, we furthermore demonstrate that the influence of these vacancies on the optical properties of the nanocrystals is determined. Since our methodology is applicable to a variety of crystalline nanostructured materials, it is expected to provide unique insights concerning structure–property correlations.

## Abstract (web summary)

CuTe nanocrystals may be used as an alternative to noble metals for plasmonics but requires understanding of the atomic structure and the influence of defects. Here Willhammar *et al*. use electron tomography to reveal the distribution of vacancies in the nanocrystals and their effect on the optical properties.

Although nanoplasmonics has been mostly restricted to the use of noble metal nanocrystals, several concerns regarding optical losses have promoted the study of a variety of alternative plasmonic (nano)materials, including copper-deficient copper chalcogenides^1^. Most studies were carried out with copper sulfides and selenides, but plasmon tunability has also been demonstrated on copper telluride nanocrystals, not only via carrier density but also via size and morphology control^2^. Interestingly, the Cu:Te ratio has been reported to affect the crystal structure. Indeed, the complex Cu–Te phase diagram reveals several phases such as orthorhombic CuTe, hexagonal Cu_2_Te and Cu_3_Te_4_ phases as well as non-stochiometric Cu_2−*x*_Te structures^3^,^4^,^5^. Recently, Li *et al*.^6^ reported that copper telluride nanocrystals, grown using a hot injection-like method, displayed a novel crystalline phase yielding interesting plasmonic properties towards sensing applications. Further progress in the optimization of the physical properties of the material requires an accurate determination of the local structure of the compound, which however has remained unknown so far because of its complexity.

Structure determination of complex nanomaterials is far from straightforward and is one of the main challenges in the field of crystallography. In addition, such studies become increasingly demanding for materials that lack perfect ordering or display disorder^7^,^8^. For X-ray and neutron diffraction, total scattering methods and pair distribution function theories are exploited to study structures containing correlated disorder^9^. For submicrometre-sized crystals, electron crystallography is often used and recent progress was initiated by the development of three-dimensional (3D) electron diffraction methods, such as rotation electron diffraction (RED)^10^ and automated diffraction tomography^11^. These methods were successfully applied to unravel the structure of a broad variety of materials, including zeolites^12^,^13^,^14^, metal organic frameworks^15^ and complex alloys^16^. Although these techniques are at the forefront of structure characterization, major progress, especially for nanocrystals, is still required.

Over the last decades, real-space electron tomography has evolved into a standard technique to investigate the morphology of nanomaterials^17^. The technique is based on the combination of multiple two-dimensional (2D) transmission electron microscopy (TEM) images acquired along different tilt angles. Recently, the quality of the 3D reconstructions has been significantly improved through the use of advanced reconstruction algorithms and, as a result, 3D imaging with atomic resolution is now possible^18^,^19^,^20^,^21^, thereby allowing monitoring and quantification of effects such as surface relaxation or strain in nanoparticles^22^. However, most of these atomic-scale 3D studies have so far been performed on noble metal nanoparticles, featuring a known and simple crystal structure.

We report here the solution of a much more challenging problem: the crystal structure of defective copper telluride nanocrystals using a combination of electron diffraction tomography and high-resolution real-space tomography. This combination provides an exciting insight into the distribution of Cu vacancies within the framework of Te atoms. The characterization was complemented by means of energy-dispersive X-ray spectroscopy (EDX) and electron energy loss spectroscopy (EELS). The outcome of our experiments was used as a realistic input model for *ab initio* calculations that enabled us to establish the connection between the atomic structure and the optical properties of this material. Our methodology is furthermore applicable to investigate a broad range of nanomaterials.

## Results

### Synthesis

The preparation of copper telluride nanoplates was based on the method reported by Li *et al*.^6^, who demonstrated the synthesis of nanocubes, nanorods and nanoplates of a novel copper telluride phase with the aid of the moisture-sensitive compound LiN(Si(CH_3_)_3_)_2_. Although the reported synthesis involved two ‘hot injection' steps and the subsequent addition of oleic acid at 70 °C during cooling to replace the weakly bound oleylamine, we obtained similar nanostructures through a one-pot approach; powder X-ray diffraction pattern (PXRD) confirmed that the phase of the obtained nanocrystals was the same as previously reported. The only pre-synthetic step in our synthesis involved the preparation of the trioctylphosphine telluride (TOP)–Te precursor. This is required since elemental tellurium is not soluble in oleylamine or oleic acid. The most important synthetic novelty is the simplicity of our approach: it is a simple ‘heat-up' protocol, does not involve hot-injections of the precursors, is highly reproducible and can be scaled up by a factor of *ca.* 5. All reagents were mixed at room temperature and subsequently heated at 200 °C for 20 min under Ar atmosphere (see Methods section for further details).

### Structural characterization

**Figure 1:**
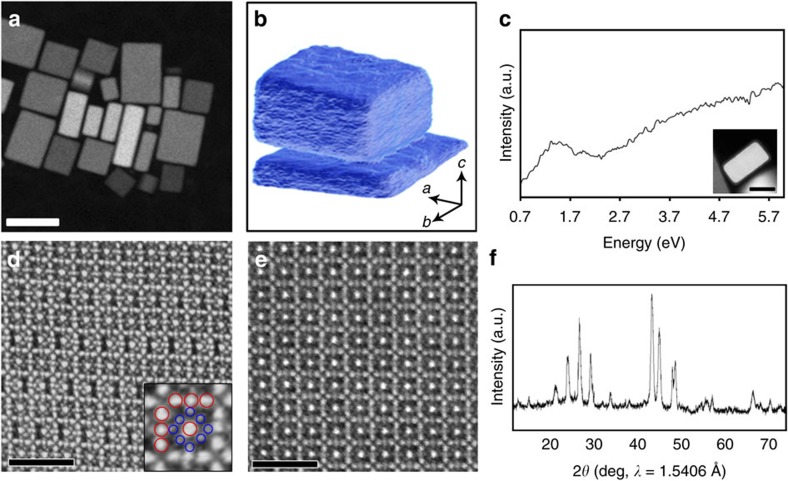
Characterization of the CuTe nanocrystals by electron microscopy methods. (**a**) HAADF-STEM overview showing the regular rectangular morphology of the particles. Scale bar, 50 nm. (**b**) Electron tomography reveals the cuboid morphology in three dimensions, with one dimension significantly shorter than the other two. The longer dimensions of the particles, *a* and *b*, are ∼25–40 nm and the shorter, *c*, corresponds to ∼15–20 nm. (**c**) EEL spectrum obtained from the low loss region after background subtraction showing a plasmon peak in the range of 1.1–1.7 eV. A HAADF-STEM image of the particle from which the data were collected is shown as inset. Scale bar, 20 nm. (**d**) High-resolution HAADF-STEM image acquired along the *a* direction. The projected distribution of heavier Te and lighter Cu atoms can be clearly observed. As an inset, the positions of Te (red) and Cu (blue) are marked. A modulation at the Cu sites can be clearly observed. The image was acquired with a long dwell time and hence sample drift gives rise to a slight geometric distortion. Scale bar, 2 nm. (**e**) High-resolution HAADF-STEM image acquired along the *c* direction does not show any obvious Cu-site ordering. (**f**) Powder X-ray diffraction pattern from the CuTe phase containing broad peaks.

A high-angle annular dark field scanning transmission electron microscopy (HAADF-STEM) overview of the resulting CuTe nanocrystals is shown in Fig. 1a. Using this technique, the image intensity scales with the atomic number *Z* of the elements present in the sample as well as with sample thickness. To investigate the morphology of the nanocrystals, a tilt series of HAADF-STEM images was acquired over a tilt range of ±72° and used as input for a 3D reconstruction algorithm^23^. It is clear that the particles yield a cuboid morphology with two longer dimensions, referred to as *a* and *b*, and a third shorter dimension called *c*, see Fig. 1b. Interestingly, EELS measurements in the low-loss region demonstrate a plasmon resonance in the range of 1.1–1.7 eV (Fig. 1c and Supplementary Fig. 1). These results confirm the plasmonic activity as previously reported^6^.

A high-resolution HAADF-STEM image was acquired along one of the long directions, *a*, of the cuboid particle, see Fig. 1d. In this image, the brighter dots (indicated by red circles) correspond to Te (*Z*=52) positions, whereas the less bright positions indicated by blue circles correspond to columns of Cu atoms (*Z*=29). The pattern in the figure indicates a complex, modulated structure, suggesting the presence of vacancies at the Cu positions. An image acquired along the shorter direction, *c*, of the cuboid (Fig. 1e) does not show this modulation. To determine the structure of the nanocrystals, PXRD was applied. However, the pattern contains broad peaks, as can be seen in Fig. 1f, preventing direct structure determination from the data.

**Figure 2:**
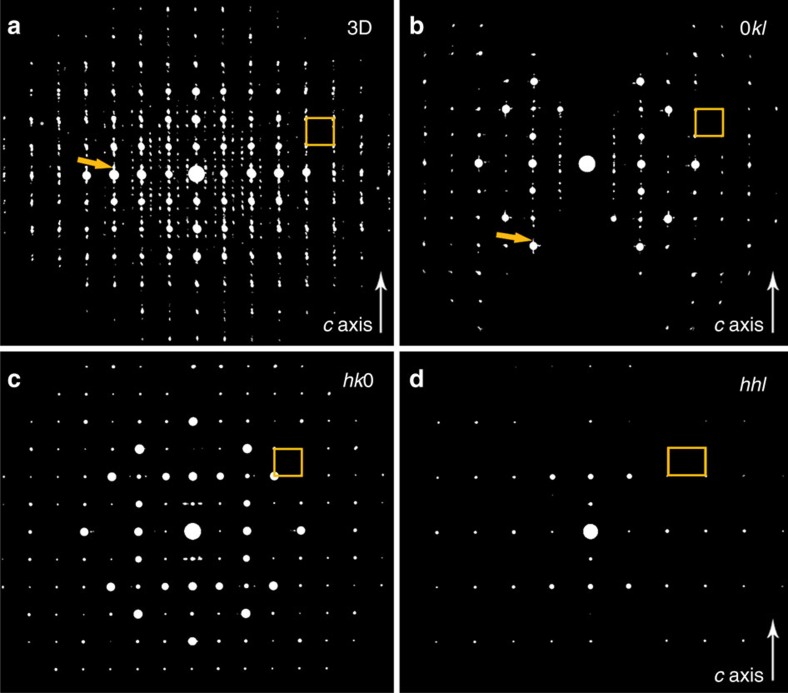
Three-dimensional electron diffraction data. (**a**) Visualization of the reconstructed 3D reciprocal lattice obtained from a representative CuTe nanocrystal. The pattern contains stronger reflections, which can be indexed in a primitive cubic unit cell with a cell parameter of 7.51 Å. In addition, weaker superstructure reflections can be observed with a periodicity of four times in the vertical direction and three times in the horizontal direction. In addition, diffusely scattered intensities are observed in between some reflections, marked by orange arrows in **a**,**b**. From the reconstructed 3D reciprocal lattice 2D sections can be visualized. (**b**) In the 0*kl* section reflections from the four times superstructure can be observed. (**c**,**d**) The *hk*0 and *hhl* sections reveal systematic extinctions for reflections with odd *h* within the 0*h*0 row and reflections with odd *l* indices with in the *hhl* section. The projected unit cell is shown by an orange rectangle in each of the panels.

**Figure 3:**
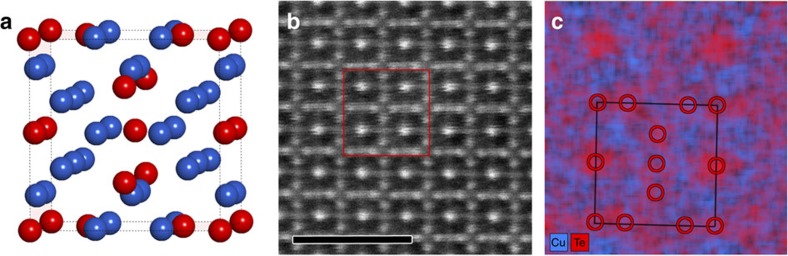
The CuTe structure model and high-resolution EDX mapping. (**a**) Average cubic structure model obtained from the electron diffraction data (Te atoms in red, Cu atoms in blue). (**b**) HAADF-STEM image acquired along the [001] direction. The red square indicates the area for which an EDX map was collected. Scale bar, 2 nm. (**c**) EDX map showing good agreement for the Te sublattice with the structure model obtained by electron diffraction.

To investigate the atomic structure in more detail, a tilt series of selected area electron diffraction (SAED) patterns was acquired from a CuTe nanocrystal over a tilt range of −62.9°/+64.2° with an increment of 0.1°. More information on the experimental conditions can be found in the Methods section. Next, this tilt series was combined into a 3D reconstruction of the reciprocal lattice, as shown in Fig. 2a. The 3D reciprocal lattice yields a combination of weak and strong reflections. The strong-intensity reflections can be indexed in a cubic unit cell with an average cell parameter of 7.51 Å. These results are in good agreement with Fig. 1d,e, where this unit cell can indeed be used to describe the contrast related to the Te lattice, observed along the [100] and [001] directions. An analysis of the reconstructed diffracted intensities suggests systematic absences for reflections within the (*hhl*) and (00*l*) groups with odd *l* indices. This leaves two possible space groups for the average structure: *Pm*-3*n* (No. 223) and *P*-43*n* (No. 218). *Ab initio* structure determination using direct methods in the space group *Pm*-3*n* resulted in a basic structure yielding eight Te atoms and 24 possible sites for Cu in one unit cell, which is illustrated in Fig. 3a; for crystallographic details see Supplementary Table 1 and Supplementary Note 1. Both space groups *Pm*-3*n* and *P*-43*n* gave equivalent solutions; hence, the higher-symmetry space group *Pm*-3*n* was selected. To the best of our knowledge this structure is a novel phase within the Cu–Te system and it is not isostructural to any other crystalline phase with related composition.

Quantitative EDX measurements, presented in Supplementary Fig. 2 and Supplementary Table 2, show that the actual Te:Cu ratio equals 1:1.5, meaning that only half of the possible Cu positions in the average crystal structure are expected to be filled. A refinement based on the electron diffraction data was performed and converged to an *R*-value of 12.94%. All Cu atoms are coordinated by four Te atoms with tetrahedral geometry. All Te atoms are coordinated by 12 Cu atoms, each of them with 0.5 occupancy, yielding an average coordination number of 6. The bond distances are in the range 2.51–2.70 Å. The coordination as well as bond distances are well in accordance with other known CuTe phases^24^,^25^,^26^. A high-resolution EDX map, see Fig. 3c, was acquired from the region indicated in Fig. 3b. It can be seen that the distribution of Te is in good agreement with the average structure model obtained by 3D electron diffraction. The distribution of Cu will be discussed in more detail below.

In addition to the stronger reflections used for structure determination, weaker reflections can be observed. The reflections can be indexed using a superstructure with a four times larger unit cell along the *c* direction, the shorter direction of the particle, and three times longer along the *a* direction, see Fig. 2a. The modulation along the *c* axis often occurs as one reflection on each side of the stronger reflections of the substructure. In between the reflections, diffusely scattered intensities can be observed, indicating disorder along the *c* axis, see Fig. 2a,b. The three times modulation is significantly weaker compared to the modulation along the *c* direction and is consistent with observations by Tu *et al*.^27^.

**Figure 4:**
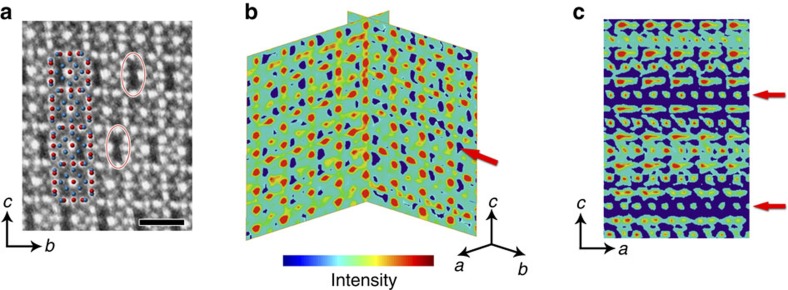
Revealing the local structure of the vacancy distribution in two- and three-dimensions. (**a**) HAADF-STEM image acquired along the [100] direction. The average structure model, including all possible Cu sites is superimposed with Te in red and Cu in blue. Some of the Cu-deficient domains are marked with red ellipses. Scale bar, 1 nm. (**b**) Perpendicular orthoslices through the electron tomography reconstruction perpendicular to the [100] and [010] directions reveal the inhomogeneous distribution of vacancies in 3D. The Te atoms are high-intensity (red) dots. In between the Te atoms, regions with a lower intensity (blue) show subvolumes inside the material with Cu deficiency (indicated by a red arrow). Long-range order of the Cu vacancies is present along the *c* axis. (**c**) An orthoslice sectioned perpendicular to the [010] direction reveals two ‘channels' of Cu deficiencies running along the *a* direction (marked by red arrows). In all panels the shorter dimension of the nanocrystal, c, is oriented along the vertical direction.

The results presented above demonstrate that the combination of electron diffraction tomography and EDX yields some first insights in the average structure of the Cu–Te nanocrystals. However, more precise details on the complex distribution of the vacancies require the use of suitable imaging methods. In the HAADF-STEM images acquired along the [100] direction the contrast distribution in between the Te atoms is clearly inhomogeneous (Fig. 4a). An overlay with the average structure clearly shows fluctuations at the Cu positions. The image shows columns of the lighter Cu atoms, in accordance with the coordinates obtained from electron diffraction. However some of the Cu positions appear black; indicating vacant positions. The ordering of the Cu is clearly visible when the nanocrystals are imaged along either of the two longer dimensions, *a* or *b*. In order to determine the 3D distribution of the vacancies, high-resolution electron tomography is required. Although this technique was already introduced as a suitable method to investigate model-like and stable (noble metal) nanoparticles^18^,^19^,^20^,^21^, the determination of the vacancy distributions in a disordered structure is still highly challenging.

We carried out a tomographic reconstruction based on five high-resolution HAADF-STEM images acquired with the rotation axis oriented along the short dimension of the cuboid nanocrystal, see the projection images in Supplementary Fig. 3. These five images were used as input for a tomographic reconstruction based on compressed sensing^28^. From the resulting tomographic reconstruction, the heavier Te atoms were found to be in good agreement with the atomic positions determined from 3D electron diffraction (Supplementary Fig. 4). However, the reconstruction does not yield any information concerning the presence of vacancies and is dominated by the intensities of the heavier Te atoms. In order to overcome this limitation, the positions of the Te atoms in one unit cell, as determined by 3D electron diffraction, were used as a template, which was fitted to the 3D reconstruction using the compressed sensing algorithm. Next, a continuous reconstruction algorithm was alternated with this template-matching method. Since the positions of the Te atoms can be refined using this procedure, the iterative coupling with the projection data leads to a gradually improving 3D reconstruction of the Cu distribution in between the Te atoms.

Orthoslices through the reconstruction, perpendicular to the [100] and [010] directions, are presented in Fig. 4b and enable a visualization of the distribution of vacancies in 3D. In this figure the low-intensity regions (blue) in between the Te atoms show the distribution of the Cu deficiencies. At this local scale, ordering along the shorter *c* dimension of the nanocrystal is clearly observed. The order of the vacancies has a preference for a modulation of four average unit cells along the *c* axis. This behaviour is further confirmed by a Fourier transform calculated from images acquired along the [100] direction (see Supplementary Fig. 5). This suggests that the superstructure can be described using a unit cell of 7.51 Å × 7.51 Å × 30.04 Å. The ordering along one of the main crystallographic directions further explains the fact that the nanocrystals exhibit a non-isotropic cuboid morphology with one dimension different from the other two. As indicated in Fig. 4c, our results show that in local parts of the nanocrystal neighbouring Cu sites are absent. This behaviour creates ‘channels' of vacancies running through the nanocrystals along two crystallographic directions: [100] and [010]. This is in agreement with the contrast observed in 2D HAADF-STEM images of Figs 1d and 4a.

### Structure–property correlation

It is important to understand the correlation between structure and optical properties, including the effect of vacancies. Therefore, calculations of the dielectric properties were performed. The dielectric properties of the *Pm*-3*n* structure with all 24 Cu sites occupied were compared to the properties of the 7.51 Å × 7.51 Å × 30.04 Å superstructure created by the presence of Cu vacancies. The distribution of the Cu vacancies is based on the findings from the electron tomography reconstruction, where vacant Cu sites form ‘channels' running through the nanocrystals. The remaining Cu vacancies that are not part of a ‘channel' were assigned according to the principle that each of the subunits follows the given stoichiometry with a Cu/Te ratio of 1.5. It should be noted that different configurations for the remaining 24 vacancies are possible. However, additional calculations (Supplementary Fig. 6) indicate that the positions of the remaining vacancies do not have a predominant effect on the optical properties.

**Figure 5:**
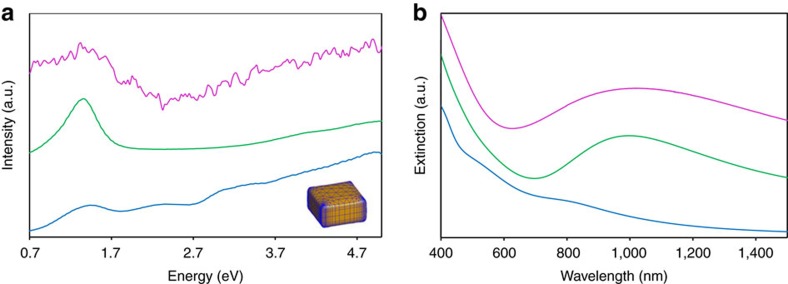
Optical properties of the material. (**a**) Simulated electron energy loss spectrum calculated from the structure model with half of the Cu sites vacant (green) shows a distinct maximum, as compared to the calculated activity based on the structure with all Cu positions occupied (blue). The experimental EEL spectrum after subtraction of the carbon background is shown for comparison in red. As an inset, a visualization of the morphology of the particle used for the simulation is shown. (**b**) Corresponding calculation of the optical extinction spectra in the Vis–NIR region shows that the structure with vacancies (green) has a significantly improved optical response compared to the model without vacancies (blue). The experimental spectrum is shown in red.

The dielectric function, 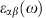 of the structure with and without Cu vacancies, was obtained from first-principles calculations within the density functional theory formalism as explained in the Methods section. In order to evaluate the importance of the vacancies, simulations of electron energy loss spectra based on the calculated dielectric properties were performed for cuboid-shaped particles with a size of 25 × 25 × 15 nm^3^ for both the structure with all Cu sites occupied and for a structure with half of the sites vacant. Whereas the energy loss spectrum from the structure without vacancies does not show any distinct maximum in the region between 0 and 2 eV, the model including vacancies predicts a distinct maximum in the low-loss function with a maximum around 1.35 eV (see Fig. 5a). This activity is consistent with the experimental EEL spectrum (Fig. 5a) that shows a band in the range between 1.1 and 1.7 eV. We additionally simulated the optical absorbance spectrum using the calculated dielectric properties, for cuboid-shaped particles with a size of 25 × 25 × 15 nm^3^ dispersed in toluene. The calculated spectrum, with a maximum ∼1,005 nm, is in good agreement with the experimental one (maximum around 1,025 nm; Fig. 5b). The agreement between the dielectric function calculations and the experiments clearly demonstrates that the presence of the vacancies determines the optical properties of the nanomaterial.

## Discussion

In summary, this work presents an accurate determination of the average structure of a previously unknown CuTe phase in a nanocrystalline form based on 3D electron diffraction data. A detailed study of the atomic structure using HAADF-STEM tomography revealed the presence of ‘channels' of vacancies running through the nanocrystals. The obtained structure was used to calculate the corresponding optical properties, which were found to be in good agreement with experimental data obtained from real nanocrystals. These results not only explain the plasmonic properties of copper-deficient copper chalcogenides, but also demonstrate the application of atomic resolution tomography to reveal an unknown crystallographic structure of complex nanocrystals containing defects.

## Methods

### Materials

Chloroform (99.8%), TOP (90%), tellurium powder (99.997%), lithium bis(trimethylsilyl) amide (LiN(SiMe_3_)_2_, 97%) and copper (II) acetate (98%) were purchased from Sigma-Aldrich. Oleylamine (80–90%) was provided from Acros Organics. Oleic acid (99%) and toluene (99%) were from Alfa-Aesar. All reactants were used as received without further purification.

### Synthesis of CuTe nanocrystals

All steps of the synthesis were implemented using the standard Schlenk techniques (Ar-filled glove box, vacuum-line, Schlenk tubes) and Fischer-Porter bottles. This permitted to apply inert atmosphere during synthesis but also for previous handling of the reagents and final storage of the products. In a typical synthesis, a soluble form of tellurium was prepared by mixing 152 mg of Te with 5 g of trioctylphosphine and heating at 150 °C under Ar for 1 h. The yellow TOP–Te solution was stirred at room temperature for 3–4 h. At a Fischer-Porter bottle containing 10 g oleylamine (80–90%) and 1.8 g oleic acid, 240 mg of Cu acetate was added. The TOP–Te solution was then transferred into the reaction mixture. An amount of 200 mg of LiN(SiMe_3_)_2_ was added, and finally the bottle filled with Ar gas was placed in an oil bath at 200 °C for 20 min under stirring (700 r.p.m.), resulting in a dark brown colour. The solution was allowed to cool down to ambient temperature under inert atmosphere. The particles were collected by centrifugation with excess chloroform/acetone (1:1 volume ratio) at a speed of 6,000 r.p.m. for 6 min. The product was finally stored in toluene.

### Material characterization

The PXRD pattern was measured using a Panalytical X'Pert PRO diffractometer with Cu-Kα radiation (1.54059 Å). Optical characterization was performed with an ultraviolet–visible–near-infrared (UV–Vis–NIR) Carry 5000 spectrophotometer using colloidal CuTe dispersions in toluene.

### Electron microscopy

Electron diffraction data were collected using a JEOL 2100 transmission electron microscope operated at 200 kV equipped with a Gatan Orius camera optimized for acquisition of diffraction patterns. The sample was mounted on a dual-axis tomography holder (Fishione 2040). The sample used for HAADF-STEM imaging, electron tomography and EELS was prepared by first adding a droplet of the CuTe colloid in toluene onto a Cu grid covered with a thin carbon film. The grid was then kept in vacuum at an elevated temperature (120 °C) for 1 h to evaporate residual organic content. HAADF-STEM imaging was performed using an aberration-corrected cubed FEI Titan operated at an acceleration voltage of 300 kV. For the tomography experiment, again a dual-axis tomography holder (Fischione 2040) was used. In order to minimize the geometrical distortions due to drifting, a series of 20 HAADF-STEM images was collected and aligned by cross-correlation before summation. EDX measurements were performed using a probe-corrected cubed FEI Titan equipped with a Super-X EDX detector system. A Mo grid was used in order to enable a quantification of the composition. EELS data were acquired using a cubed FEI Titan equipped with a monochromator operated at 120 kV. The EELS data from the particle and the carbon support were normalized based on the intensity of the zero-loss peak.

### Crystallographic analysis

Electron diffraction data were collected using the RED method in order to obtain a 3D data set with fine sampling of reciprocal space. A goniometer tilt step of 2° was complimented by a finer beam tilt step of 0.1° in order to obtain a data set continuously sampling reciprocal space with a 0.1° step. The reconstruction from the tilt series of SAED patterns and the extraction of diffracted intensities were performed using the RED software^29^. Symmetry deduction was performed based on systematic extinctions. Reflection conditions were found to be *hhl l*=2n and 00l* l*=2n. Some weaker reflections were found to violate the conditions; this can both be due to multiple interaction as the particle passed close to the [001] zone axis during data collection as well as the presence of diffusely scattered intensities. The reflection conditions leave two possible space groups *Pm*-3*n* (No. 223) and *P*-43*n* (No. 218). The structure determination and refinement from the reconstructed reciprocal lattice were performed using direct methods implemented in the software SIR2011 (ref. 30). The higher-symmetry space group *Pm*-3*n* was selected as the sub-symmetry space group *P*-43*n* gave an equivalent result after structure solution and refinement. The internal symmetry residual (Rint) for the electron diffraction data in space group *Pm-3n* is 0.187 up to a resolution of 0.85 Å. The least squares refinement of the structure resulted in a fit with good figures of merit, *R*=0.129 and a goodness of fit of 1.56. Structure determination based on data obtained with by an alternative experimental set-up, using only goniometer tilt, resulted in an identical structure model.

### Electron tomography

High-resolution electron tomography reconstruction was based on five zone axis HAADF-STEM images. Images were acquired along [100], [210] (26°), [110] (45°), [120] (64°) and [010] (90°) directions with tilt angles relative to the first zone axis in parentheses. For each orientation, 20 images acquired with a relatively short dwell time of 1 μs were summed up after cross-correlation in order to reduce the effect of sample drift during acquisition. Five projection images were used as an input for a tomographic reconstruction using a compressed sensing algorithm developed in earlier work^28^. From the resulting tomographic reconstruction, the heavier Te atoms were found to be in good agreement with the atomic positions determined from 3D electron diffraction. In order to improve the reconstruction, the positions of the Te atoms in one unit cell, as determined by 3D electron diffraction were used as a template, which was fitted to the 3D reconstruction using the compressed sensing algorithm. This results in a 3D similarity map in which a voxel with a value close to 1 is likely to indicate the centre of a unit cell. A threshold of 0.6 was used to determine the positions of the unit cells only containing the Te atoms. In this manner, a list with the estimated 3D coordinates of the Te atoms is derived and for each position, a 3D Gaussian is added to the reconstruction. Next, a continuous reconstruction algorithm is alternated with this template-matching method. Since the positions of the Te atoms can be refined using this procedure, the iterative coupling with the projection data will lead to a gradually improving 3D reconstruction of the distribution of Cu atoms.

### Computational details

The quantity accessible to first-principles calculations is the dielectric function, 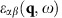, which is in general a 3 × 3  2nd rank tensor. The optical absorption and the electron energy loss are directly obtained from 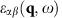. We evaluated the dielectric function in the independent-particle approximation for *q*→0 within the density functional theory formalism as implemented in the Vienna *ab initio* simulation package^31^. The projector augmented wave method was applied, where the Cu (3d^10^4s^1^) and Te (5s^2^5p^4^) electrons are treated as valence electrons. The exchange-correlation functional was calculated using the generalized gradient approximation of Perdew–Burke–Ernzerhof 32. A 12 × 12 × 4 Monkhorst-Pack mesh was chosen to sample the first Brillouin zone and the energy cutoff of the plane wave basis set was set at 300 eV. For further details on the calculations see the Supplementary Methods and Supplementary Figs 7–11. Once we have obtained the dielectric function, the extinction spectrum is calculated for a cuboid-shaped particle with a size of 25 × 25 × 15 nm^3^, dispersed in toluene with refractive index 1.4968, using the DDSCAT code^33^, which is based upon the discrete dipole approximation. The EEL spectrum was simulated for a particle with a cuboid morphology and size of 25 × 25 × 15 nm^3^ using the MATLAB toolbox MNPBEM^34^.

### Data availability

Details regarding the presented crystal structure is available in CIF format in the Supplementary Information. Other data that support the findings of this study are available from the corresponding author upon request.

## Additional information

**How to cite this article:** Willhammar, T. *et al*. Structure and vacancy distribution in copper telluride nanoparticles influence plasmonic activity in the near-infrared. *Nat. Commun.* **8,** 14925 doi: 10.1038/ncomms14925 (2017).

**Publisher's note**: Springer Nature remains neutral with regard to jurisdictional claims in published maps and institutional affiliations.

## Supplementary Material

Supplementary Information Supplementary Figures, Supplementary Tables, Supplementary Note, Supplementary Methods and Supplementary References

## References

[b1] Comin A. & Manna L. New materials for tunable plasmonic colloidal nanocrystals. Chem. Soc. Rev. 43, 3957–3975 (2014). 24435209 10.1039/c3cs60265f

[b2] Kriegel I. . Shedding light on vacancy-doped copper chalcogenides: shape-controlled synthesis, optical properties, and modeling of copper telluride nanocrystals with near-infrared plasmon resonances. ACS Nano 7, 4367–4377 (2013). 23570329 10.1021/nn400894d

[b3] Wu X. . Phase control of Cu_x_Te film and its effects on CdS/CdTe solar cell. Thin Solid Films 515, 5798–5803 (2007).

[b4] Li H. . Synthesis of uniform disk-shaped copper telluride nanocrystals and cation exchange to cadmium telluride quantum disks with stable red emission. J. Am. Chem. Soc. 135, 12270–12278 (2013). 23865842 10.1021/ja404694k

[b5] Xiao G. . Controlled synthesis of hollow Cu_2−x_ Te nanocrystals based on the Kirkendall effect and their enhanced CO gas-sensing properties. Small 9, 793–799 (2013). 23161794 10.1002/smll.201202083

[b6] Li W. . CuTe nanocrystals: shape and size control, plasmonic properties, and use as SERS probes and photothermal agents. J. Am. Chem. Soc. 135, 7098–7101 (2013). 23647089 10.1021/ja401428e

[b7] Baerlocher C., Weber T., McCusker L. B., Palatinus L. & Zones S. I. Unraveling the perplexing structure of the zeolite SSZ-57. Science 333, 1134–1137 (2011). 21868674 10.1126/science.1207466

[b8] Willhammar T. . Structure and catalytic properties of the most complex intergrown zeolite ITQ-39 determined by electron crystallography. Nat. Chem. 4, 188–194 (2012). 22354432 10.1038/nchem.1253

[b9] Keen D. A. & Goodwin A. L. The crystallography of correlated disorder. Nature 521, 303–309 (2015). 25993960 10.1038/nature14453

[b10] Zhang D., Oleynikov P., Hovmöller S. & Zou X. Collecting 3D electron diffraction data by the rotation method. Z. Kristallogr. Int. J. Struct. Phys. Chem. Asp. Cryst. Mater. 225, 94–102 (2010).

[b11] Kolb U., Gorelik T., Kübel C., Otten M. T. & Hubert D. Towards automated diffraction tomography: part I—data acquisition. Ultramicroscopy 107, 507–513 (2007). 17234347 10.1016/j.ultramic.2006.10.007

[b12] Jiang J. . Synthesis and structure determination of the hierarchical meso-microporous zeolite ITQ-43. Science 333, 1131–1134 (2011). 21868673 10.1126/science.1208652

[b13] Willhammar T., Yun Y. & Zou X. Structural determination of ordered porous solids by electron crystallography. Adv. Funct. Mater. 24, 182–199 (2014).

[b14] Guo P. . A zeolite family with expanding structural complexity and embedded isoreticular structures. Nature 524, 74–78 (2015). 26176918 10.1038/nature14575

[b15] Feyand M. . Automated diffraction tomography for the structure elucidation of twinned, sub-micrometer crystals of a highly porous, catalytically active bismuth metal–organic framework. Angew. Chem. Int. Ed. 51, 10373–10376 (2012). 10.1002/anie.201204963 22976879

[b16] Singh D. . A complex pseudo-decagonal quasicrystal approximant, Al_37_ (Co,Ni)_15.5_ , solved by rotation electron diffraction. J. Appl. Crystallogr. 47, 215–221 (2014).

[b17] Midgley P. A. & Weyland M. 3D electron microscopy in the physical sciences: the development of Z-contrast and EFTEM tomography. Ultramicroscopy 96, 413–431 (2003). 12871805 10.1016/S0304-3991(03)00105-0

[b18] Van Aert S., Batenburg K. J., Rossell M. D., Erni R. & Van Tendeloo G. Three-dimensional atomic imaging of crystalline nanoparticles. Nature 470, 374–377 (2011). 21289625 10.1038/nature09741

[b19] Goris B. . Atomic-scale determination of surface facets in gold nanorods. Nat. Mater. 11, 930–935 (2012). 23085569 10.1038/nmat3462

[b20] Goris B. . Three-dimensional elemental mapping at the atomic scale in bimetallic nanocrystals. Nano Lett. 13, 4236–4241 (2013). 23952010 10.1021/nl401945b

[b21] Chen C.-C. . Three-dimensional imaging of dislocations in a nanoparticle at atomic resolution. Nature 496, 74–77 (2013). 23535594 10.1038/nature12009

[b22] Goris B. . Measuring lattice strain in three dimensions through electron microscopy. Nano Lett. 15, 6996–7001 (2015). 26340328 10.1021/acs.nanolett.5b03008 PMC4877113

[b23] van Aarle W. . The ASTRA toolbox: a platform for advanced algorithm development in electron tomography. Ultramicroscopy 157, 35–47 (2015). 26057688 10.1016/j.ultramic.2015.05.002

[b24] Forman S. & Peacock M. Crystal structure of rickardite, Cu_4−x_Te_2_. Am. Mineral. 34, 441–451 (1949).

[b25] Cameron E. N. & Threadgold I. M. Vulcanite, a new copper telluride from Colorado, with notes on certain associated minerals. Am. Mineral. 46, 258–268 (1961).

[b26] Baranova R. V., Avilov A. S. & Pinsker Z. G. Determination of the crystal structure of the hexagonal phase beta III in the Cu-Te system by electron diffraction. Sov. Phys. Crystallogr. 18, 736–740 (1974).

[b27] Tu R. . Influence of the ion coordination number on cation exchange reactions with copper telluride nanocrystals. J. Am. Chem. Soc. 138, 7082–7090 (2016). 27177274 10.1021/jacs.6b02830 PMC5736242

[b28] Leary R., Saghi Z., Midgley P. A. & Holland D. J. Compressed sensing electron tomography. Ultramicroscopy 131, 70–91 (2013). 23834932 10.1016/j.ultramic.2013.03.019

[b29] Wan W., Sun J., Su J., Hovmöller S. & Zou X. Three-dimensional rotation electron diffraction: software *RED* for automated data collection and data processing. J. Appl. Crystallogr. 46, 1863–1873 (2013). 24282334 10.1107/S0021889813027714 PMC3831301

[b30] Burla M. C. . SIR2011: a new package for crystal structure determination and refinement. J. Appl. Crystallogr. 45, 357–361 (2012).

[b31] Gajdoš M., Hummer K., Kresse G., Furthmüller J. & Bechstedt F. Linear optical properties in the projector-augmented wave methodology. Phys. Rev. B 73, 045112 (2006).

[b32] Perdew J. P., Burke K. & Ernzerhof M. Generalized gradient approximation made simple. Phys. Rev. Lett. 77, 3865–3868 (1996). 10062328 10.1103/PhysRevLett.77.3865

[b33] Draine B. T. & flatau p. j. discrete-Dipole Approximation For Scattering Calculations. J. Opt. Soc. Am. A 11, 1491 (1994).

[b34] Hohenester U. & Trügler A. MNPBEM—a Matlab toolbox for the simulation of plasmonic nanoparticles. Comput. Phys. Commun. 183, 370–381 (2012).

