# Supplementary material for: Structure and vacancy distribution in copper telluride nanoparticles influence plasmonic activity in the near-infrared
Source: Nat Commun. 2017 Mar 30;8:14925. doi: 10.1038/ncomms14925 (PMC5379103; doi:10.1038/ncomms14925)
Supplement: Supplementary Information — Supplementary Figures, Supplementary Tables, Supplementary Note, Supplementary Methods and Supplementary References [file ncomms14925-s1.pdf]

# Supplementary information

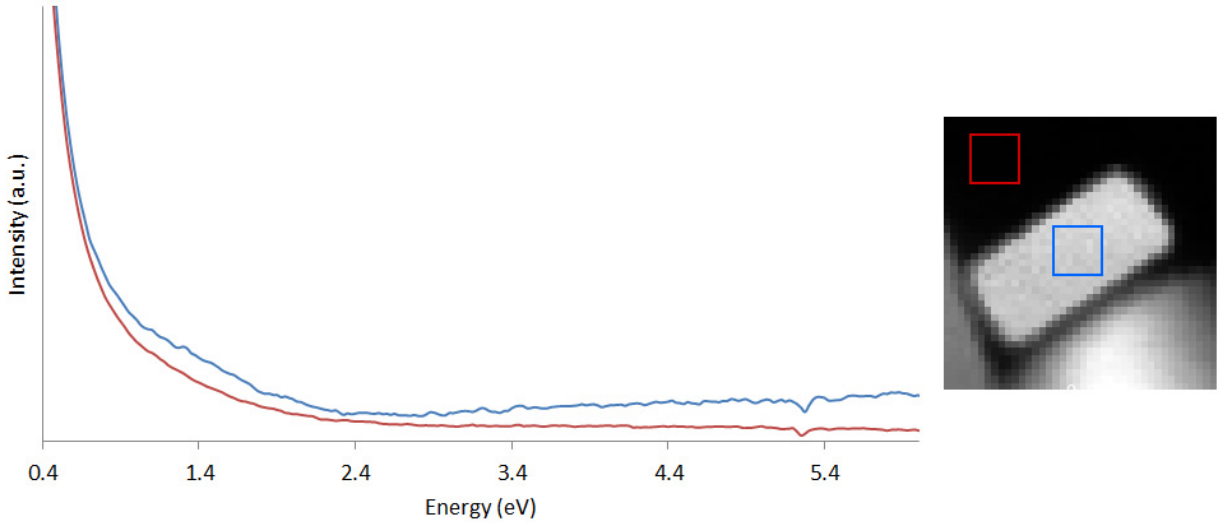


**Supplementary Figure 1.** EELS spectra from a single particle (blue) and from the amorphous carbon support (red) without background subtraction. The regions from which the spectra were obtained are indicated in the figure shown as an inset. An increased difference between the two signals can be observed in the region 1.1-1.7 eV.

**Supplementary Table 1.** Details about electron diffraction data and analysis from the structure determination and refinement.

| | |
|---|---|
| Tilt angle | -62.9° to +64.2° |
| Tilt step | 0.1° |
| No. of RED frames | 1321 |
| Exposure time/frame | 1.0 s |
| $a$ / Å | 7.47 |
| $b$ / Å | 7.65 |
| $c$ / Å | 7.42 |
| $\alpha$ / ° | 90.0 |
| $\beta$ / ° | 90.2 |
| $\gamma$ / ° | 90.2 |
| Space group | $Pm$-3$n$ |
| Resolution | 0.85 Å |
| Completeness | 100% |
| $R$int | 0.1868 |
| No. of measured reflections | 1781 |
| No. of independent reflections | 80 |
| $h$ | $-8 \leq h \leq 8$ |
| $k$ | $-7 \leq k \leq 6$ |
| $l$ | $-8 \leq l \leq 8$ |
| $R$1 | 0.1294 |

**Supplementary Note 1.** Crystallographic structure information for the average structure as determined and refined based on electron diffraction data. The structure was determined in the cubic space group *Pm*-3*n*.

data_CuTe

_audit_creation_method SIR2011

_chemical_formula_moiety 'Te1 Cu1.5'

_chemical_formula_weight 37.16

_chemical_formula_sum 'Te0.1667 Cu0.25'

_computing_structure_solution

; SIR2011 (Burla et al., 2011)

;

_computing_structure_refinement

; SIR2011 (Burla et al., 2011)

;

_computing_molecular_graphics

; SIR2011 (Burla et al., 2011)

;

_computing_publication_material

; SIR2011 (Burla et al., 2011)

;

_cell_length_a 7.510

_cell_length_b 7.510

_cell_length_c 7.510

_cell_angle_alpha 90.000

_cell_angle_beta 90.000

_cell_angle_gamma 90.000

_cell_volume 423.56

_cell_formula_units_Z 48

_cell_measurement_wavelength 0.02510

_cell_measurement_reflns_used 25

_cell_measurement_theta_min 0.135

_cell_measurement_theta_max 0.840

_cell_measurement_temperature 293

_symmetry_cell_setting 'Cubic'

_symmetry_space_group_name_H-M 'P m -3 n'

_symmetry_Int_Tables_number 223

loop_

_symmetry_equiv_pos_site_id

_symmetry_equiv_pos_as_xyz

1 'x,y,z'

2 '-x,-y,z'

3 'x,-y,-z'

4 '-x,y,-z'

5 'z,x,y'

6 'y,z,x'

7 '-z,-x,y'

8 '-y,z,-x'

9 'z,-x,-y'

10 '-y,-z,x'

11 '-z,x,-y'

12 'y,-z,-x'

13 '1/2-y,1/2-x,1/2-z'

14 '1/2+y,1/2+x,1/2-z'

15 '1/2+y,1/2-x,1/2+z'

16 '1/2-y,1/2+x,1/2+z'

17 '1/2-x,1/2-z,1/2-y'

18 '1/2-z,1/2-y,1/2-x'

19 '1/2+x,1/2+z,1/2-y'

20 '1/2-z,1/2+y,1/2+x'

21 '1/2+x,1/2-z,1/2+y'

22 '1/2+z,1/2+y,1/2-x'

23 '1/2-x,1/2+z,1/2+y'

24 '1/2+z,1/2-y,1/2+x'

25 '-x,-y,-z'

26 'x,y,-z'

27 '-x,y,z'

28 'x,-y,z'

29 '-z,-x,-y'

30 '-y,-z,-x'

31 'z,x,-y'

32 'y,-z,x'

33 '-z,x,y'

34 'y,z,-x'

35 'z,-x,y'

36 '-y,z,x'

37 '1/2+y,1/2+x,1/2+z'

38 '1/2-y,1/2-x,1/2+z'

39 '1/2-y,1/2+x,1/2-z'

40 '1/2+y,1/2-x,1/2-z'

41 '1/2+x,1/2+z,1/2+y'

42 '1/2+z,1/2+y,1/2+x'

43 '1/2-x,1/2-z,1/2+y'

44 '1/2+z,1/2-y,1/2-x'

45 '1/2-x,1/2+z,1/2-y'

46 '1/2-z,1/2-y,1/2+x'

47 '1/2+x,1/2-z,1/2-y'

48 '1/2-z,1/2+y,1/2-x'

```
_exptl_crystal_description          'cuboid nanocrystal'
_exptl_crystal_density_diffrn       6.994
_exptl_crystal_F_000                764
_exptl_absorpt_coefficient_mu       0.000

_exptl_absorpt_correction_type      'psi-scan'
_exptl_absorpt_correction_T_min     0.0000
_exptl_absorpt_correction_T_max     0.0000

_diffrn_ambient_temperature         293
_diffrn_radiation_wavelength        0.02510
_diffrn_radiation_type              electron

_diffrn_radiation_source            LaB6
_diffrn_measurement_device          JEOL_2100
_diffrn_measurement_method          RED

loop_
_diffrn_reflns_number               1781
_diffrn_reflns_av_R_equivalents     0.2563

_diffrn_reflns_theta_min            0.135
```

_diffrn_reflns_theta_max 0.840

_diffrn_reflns_theta_full 0.840

_diffrn_measured_fraction_theta_max 1.0

_diffrn_measured_fraction_theta_full 1.0

_diffrn_reflns_limit_h_max 8

_diffrn_reflns_limit_h_min 1

_diffrn_reflns_limit_k_max 6

_diffrn_reflns_limit_k_min 0

_diffrn_reflns_limit_l_max 4

_diffrn_reflns_limit_l_min 0

loop_

_atom_type_symbol

_atom_type_number_in_cell

_atom_type_description

_atom_type_scat_dispersion_real

_atom_type_scat_dispersion_imag

_atom_type_scat_source

'Cu ' 12 'Copper ' 0.0000 0.0000 ' Int. Tab. C, 4.2.6.8, 6.1.1.4'

'Te ' 8 'Tellurium ' 0.0000 0.0000 ' Int. Tab. C, 4.2.6.8, 6.1.1.4'

_reflns_number_total 80

_reflns_number_gt 74

_reflns_threshold_expression ' F >3.0 \s(F) '

_reflns_limit_h_max 8

_reflns_limit_h_min 1

_reflns_limit_k_max 6

_reflns_limit_k_min 0

_reflns_limit_l_max 4

_reflns_limit_l_min 0

_reflns_d_resolution_high 0.856

```
_reflns_d_resolution_low                5.310
_refine_ls_structure_factor_coef        F

_refine_ls_extinction_method            'none'

_refine_ls_matrix_type                  'Full'
_refine_ls_hydrogen_treatment           ?
_refine_ls_weighting_scheme             'calc'
_refine_ls_weighting_details
;    w=1.0
;
_refine_ls_number_reflns                74
_refine_ls_number_parameters            6
_refine_ls_number_restraints            0
_refine_ls_number_constraints           0
_refine_ls_R_factor_all                 0.129
_refine_ls_R_factor_gt                  0.129
_refine_ls_wR_factor_all                0.125
_refine_ls_wR_factor_gt                 0.125
_refine_ls_goodness_of_fit_all          1.598
_refine_ls_goodness_of_fit_gt           1.598
_refine_ls_shift/su_max                 0.000
_refine_ls_shift/su_mean                0.000

loop_
_atom_site_label
_atom_site_fract_x
_atom_site_fract_y
_atom_site_fract_z
_atom_site_occupancy
```

_atom_site_U_iso_or_equiv

_atom_site_thermal_displace_type

_atom_site_type_symbol

_atom_site_symmetry_multiplicity

```
TE1   0.5000     0.2500     0.0000    1.000      0.050(4) uiso te  6
TE2   1.0000     0.0000     0.0000    1.000      0.093(8) uiso te  2
CU1   0.706(2)   0.0000     0.151(2)  0.500      0.054(5) uiso cu 24
```

# Start Validation Reply Form

_vrf_RADNT01_CuTe

;

PROBLEM: The radiation type should contain one of the following

RESPONSE: Electron diffraction was performed however 'electron' is not a recognized option in CheckCIF

;

_vrf_RINTA01_CuTe

;

PROBLEM: The value of Rint is greater than 0.25

RESPONSE: R-values are higher for electron diffraction because of dynamic scattering

;

_vrf_PLAT020_CuTe

;

PROBLEM: The value of Rint is greater than 0.12

RESPONSE: R-values are higher for electron diffraction because of dynamic scattering

;

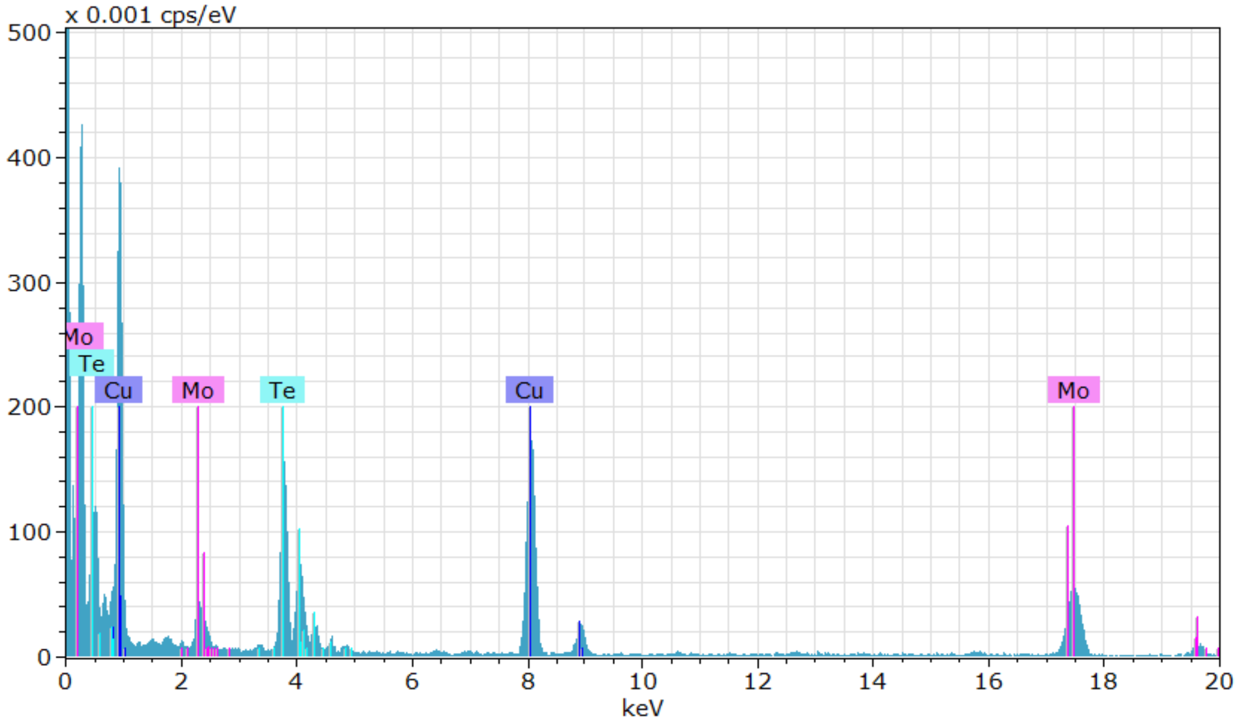


**Supplementary Figure 2.** EDX spectrum of the CuTe nanoparticles measured on a carbon-coated Mo grid.

**Supplementary Table 2.** Quantification of seven EDX measurements from CuTe nanoparticles measured on a carbon coated Mo grid. The measurements result in an average of 40.0% Te and 60.0% Cu with a standard deivation of 1.6%.

| Measurement | Cu (at %) | Te (at %) |
|---|---|---|
| 1 | 58.4 | 41.6 |
| 2 | 59.4 | 40.6 |
| 3 | 58.6 | 41.4 |
| 4 | 61.0 | 39.0 |
| 5 | 62.4 | 37.6 |
| 6 | 58.8 | 41.2 |
| 7 | 61.2 | 38.8 |
| Average | 59.97 | 40.03 |

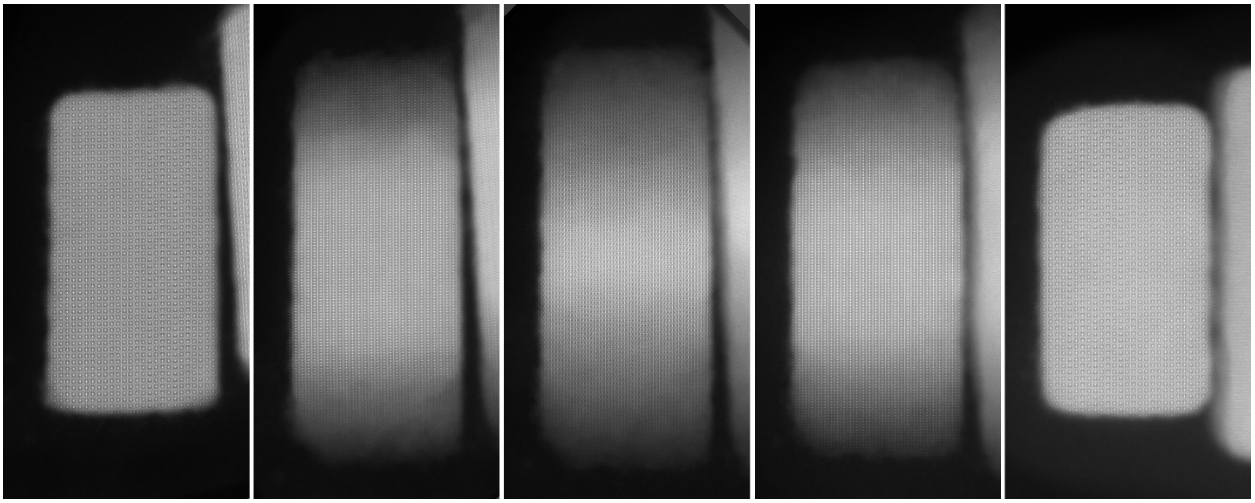

**Supplementary Figure 3.** The 5 HAADF-STEM images used for the atomic resolution tomography reconstruction. Each of the images is a summation of 20 images in order to reduce distortions due to sample drift.

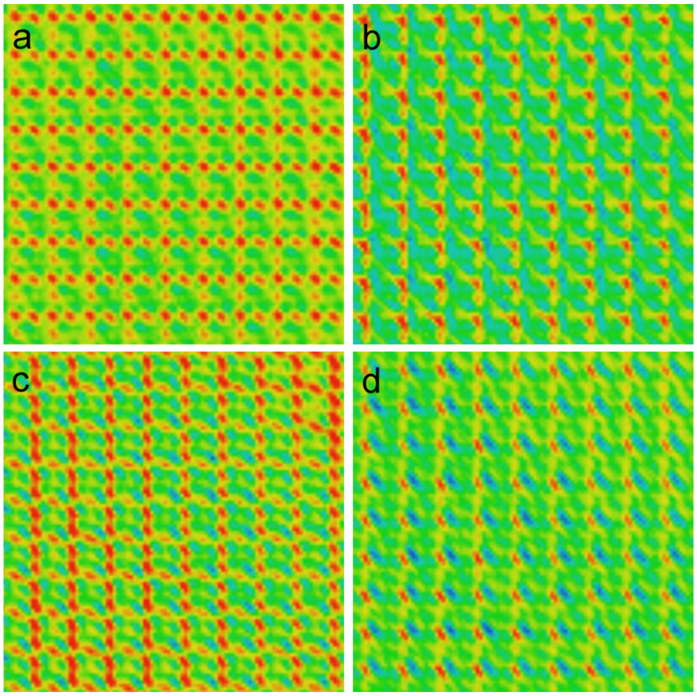


**Supplementary Figure 4.** Orthoslices perpendicular to the [001] direction through the initial electron tomography reconstruction performed by compressed sensing. The orthoslices were obtained at an offset of (a) 0 (b) 0.25 (c) 0.5 and (d) 0.75 multiples of the unit cell (7.51 Å). Red contrast is the highest intensity and resembles the positions of the heavier Te atoms as they were determined from electron diffraction.

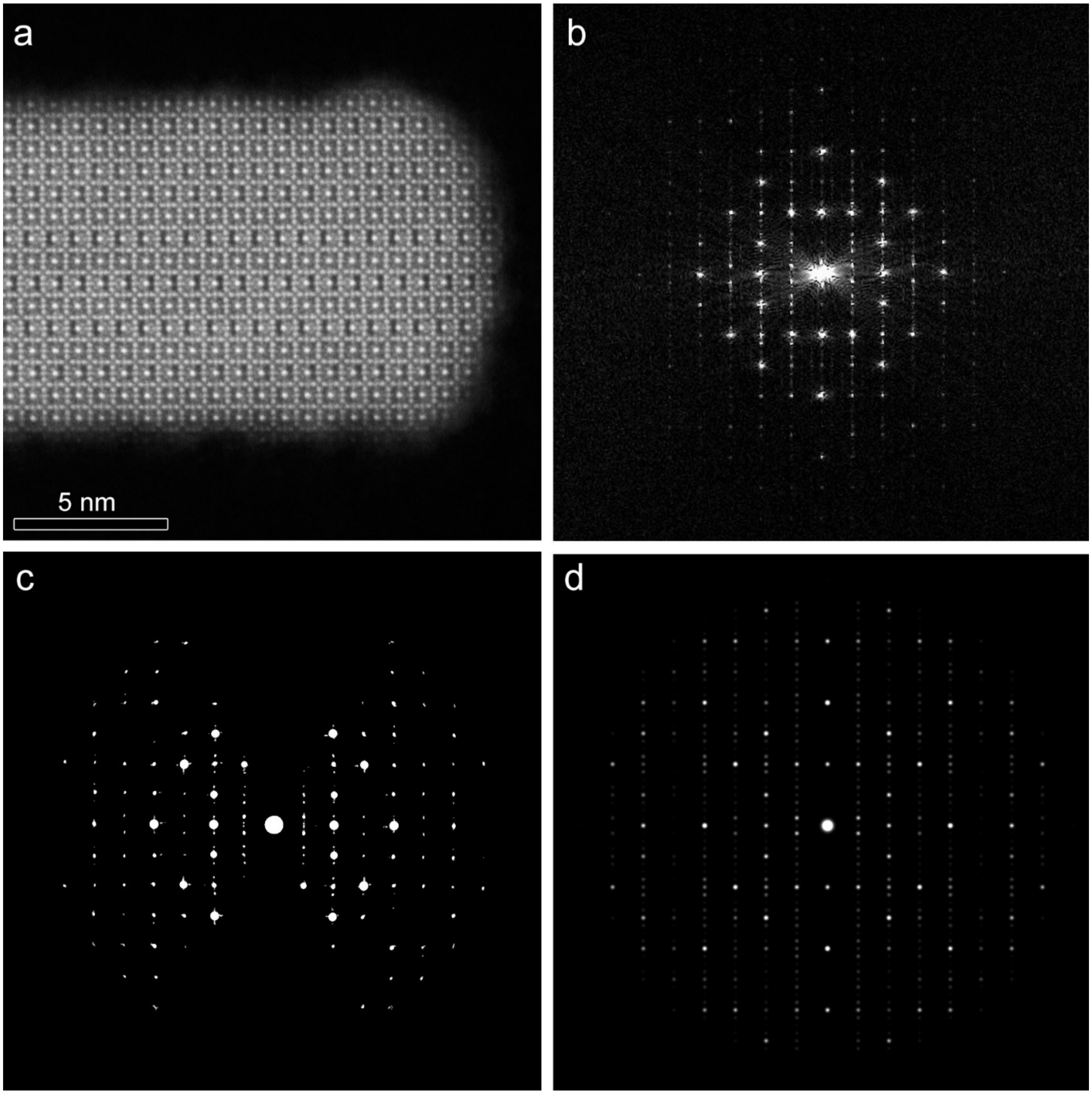


**Supplementary Figure 5.** (a) HAADF-STEM image from a CuTe particle aligned along the [100] direction. (b) The corresponding Fourier transform calculated from (a). (c) Section from the reconstructed 3D reciprocal lattice perpendicular to the [001] direction and (d) simulated kinematical electron diffraction pattern along the same direction based on the structure including vacancies. In the Fourier transform a modulation can be observed running along the vertical ***c*** direction. In (a) the corresponding modulation of four times of the average unit cell along the ***c***

direction can be observed in some parts of the particle. The electron diffraction data as well as the simulated diffraction pattern exhibits the same modulation.

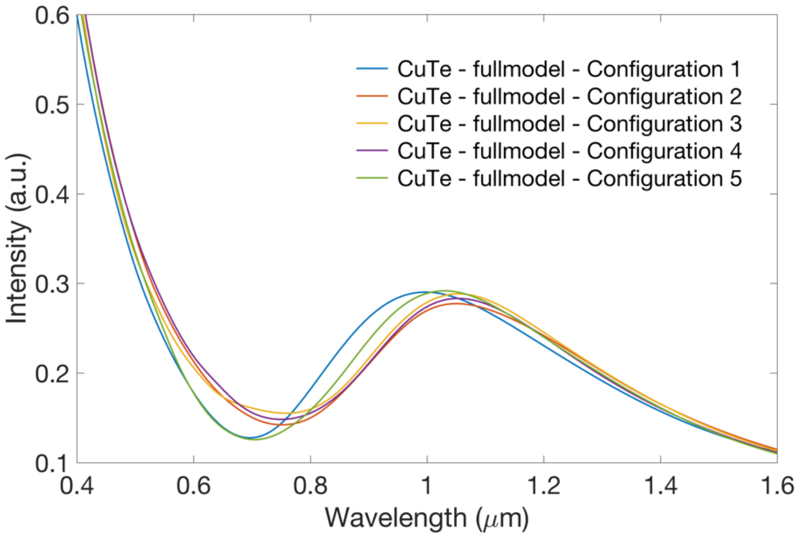


**Supplementary Figure 6.** Calculations of the extinction spectrum for five different configurations of the 24 Cu vacancies remaining undecided after analysis of the electron tomography results.

**Supplementary Methods.** Dielectric properties from first principles.

The dielectric function, which is in general a 3x3 second rank tensor, can be written as the sum of the inter- and intraband contributions[2]:

$$\varepsilon_{\alpha\beta}(\omega) = \varepsilon_{\alpha\beta}^{\text{inter}}(\omega) + \varepsilon_{\alpha\beta}^{\text{intra}}(\omega). \qquad (1)$$

Writing $\varepsilon_{\alpha\beta}(\omega) = \varepsilon_{\alpha\beta}^{(1)}(\omega) + i\varepsilon_{\alpha\beta}^{(2)}(\omega)$, the imaginary part of the interband dielectric tensor is directly calculated from the transition matrix elements within the random phase approximation and the long-wavelength limit ($\mathbf{q} \to 0$):

$$\varepsilon_{\alpha\beta}^{(2),\text{inter}}(\omega) = \frac{4\pi^2 e^2}{\Omega} \lim_{q\to 0} \frac{1}{q^2} \sum_{\mathrm{c,v},\mathbf{k}} 2f_{\mathrm{v}\mathbf{k}}\, g_{\mathbf{k}} \langle u_{\mathrm{c}\mathbf{k}+\mathbf{e}_\alpha q} | u_{\mathrm{v}\mathbf{k}} \rangle \langle u_{\mathrm{c}\mathbf{k}+\mathbf{e}_\beta q} | u_{\mathrm{v}\mathbf{k}} \rangle^* \, \delta(E_{\mathrm{c}\mathbf{k}} - E_{\mathrm{v}\mathbf{k}} - \hbar\omega), \quad (2)$$

where $\Omega$ is the volume of the unit cell, $\mathbf{e}_\alpha$ and $\mathbf{e}_\beta$ are the Cartesian unit vectors, $u_{\mathbf{vk}}$ and $u_{\mathbf{ck}}$ are, respectively, the cell-periodic parts of the Bloch functions of the valence and conduction band for the k-point **k**, and $E_{\mathbf{vk}}$, $E_{\mathbf{ck}}$ their corresponding Kohn-Sham eigenenergies. $f_{\mathbf{vk}} \equiv f(E_{\mathbf{vk}})$ is the occupation number and $g_{\mathbf{k}}$ the weight of the corresponding k-point. The imaginary part of the intraband dielectric tensor is calculated using the Drude model:

$$\varepsilon_{\alpha\beta}^{(2),\mathrm{intra}}(\omega) = \frac{\gamma\omega_{\alpha\beta}^2}{\omega(\omega^2+\gamma^2)}, \qquad (3)$$

where the damping parameter is set to $\gamma = 0.025$ eV, which corresponds to room temperature. The so-called intraband plasma frequency (squared) $\omega_{\alpha\beta}^2$ is calculated from first principles using the expression

$$\omega_{\alpha\beta}^2 = \frac{4\pi e^2}{\Omega\hbar^2}\sum_{\mathrm{n},\mathbf{k}} 2g_{\mathbf{k}}\frac{\partial f(E_{\mathbf{nk}})}{\partial E}\left(\mathbf{e}_\alpha\frac{\partial E_{\mathbf{nk}}}{\partial \mathbf{k}}\right)\left(\mathbf{e}_\beta\frac{\partial E_{\mathbf{nk}}}{\partial \mathbf{k}}\right). \qquad (4)$$

Finally, the real part of the dielectric tensor $\varepsilon_{\alpha\beta}^{(1)}(\omega)$ is derived from the Kramers-Kronig relation after adding the imaginary parts of the inter- and intraband dielectric function:

$$\varepsilon_{\alpha\beta}^{(1)}(\omega) = 1 + \frac{1}{\pi}P\int\frac{\varepsilon_{\alpha\beta}^{(2),\mathrm{inter}}(\omega')}{\omega'-\omega}\mathrm{d}\omega' + \frac{1}{\pi}P\int\frac{\varepsilon_{\alpha\beta}^{(2),\mathrm{intra}}(\omega')}{\omega'-\omega}\mathrm{d}\omega', \qquad (5)$$

Where $P$ is the Cauchy principal value.

For the analysis presented in this text, we have used the results of vacancy configuration 1 (See Supplementary Figure 6). Because of the symmetry of the unit cell, the first two diagonal elements of both $\varepsilon_{\alpha\beta}^{\mathrm{inter}}(\omega)$ and $\omega_{\alpha\beta}$ are equal. For the intraband plasma frequency, we find $\omega_{\mathrm{xx}} = \omega_{\mathrm{yy}} =$ 1.76 eV and $\omega_{\mathrm{zz}} = 0.90$ eV (frequencies are expressed in eV throughout the text). Since the particles are considered to have a random orientation in the solvent, we calculate the dielectric function $\varepsilon(\omega)$ by averaging the diagonal elements of the dielectric tensor $\varepsilon_{\alpha\beta}(\omega)$. In Supplementary Figure 7 we show the calculated real and imaginary part of the averaged diagonal components of the dielectric tensor, as well as its inter- and intraband contributions.

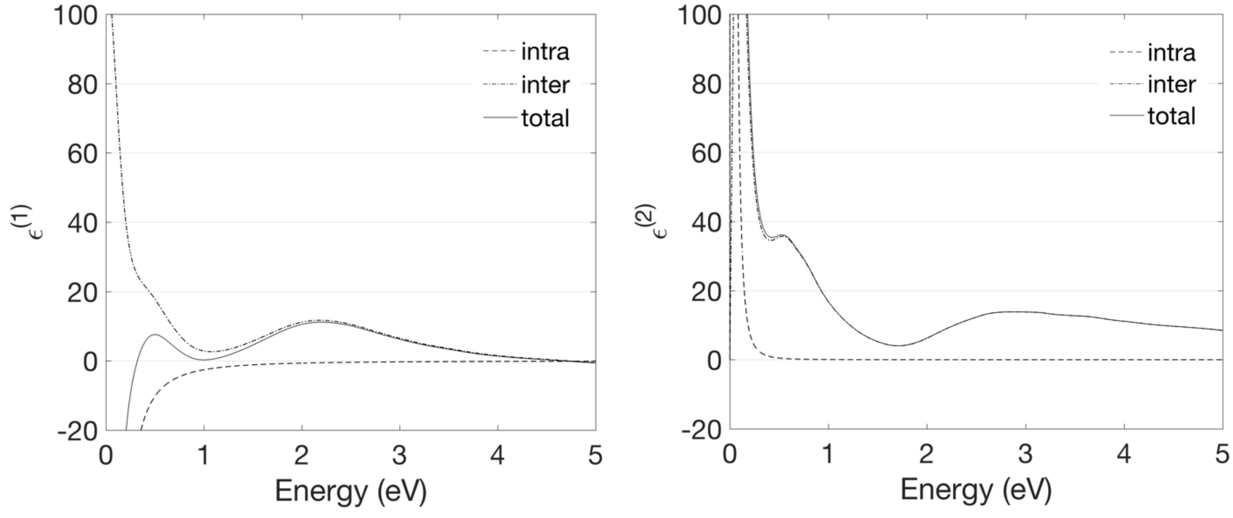


**Supplementary Figure 7.** Real (left) and imaginary (right) part of the dielectric function.

To correlate the results of the dielectric function with the optical absorption experiments, we calculate the optical absorption coefficient $\alpha(\omega) = 4\pi\omega \cdot \hat{k}(\omega)/(hc)$, where the extinction coefficient $\hat{k}(\omega)$ is given by[3]

$$\hat{k}(\omega) = \sqrt{\frac{|\varepsilon(\omega)| - \varepsilon^{(1)}(\omega)}{2}}. \qquad (5)$$

Finally, we consider a sample of thickness $L = 25$ nm to calculate the absorbance spectrum $a(\omega) = 1 - e^{-L\cdot\alpha(\omega)}$. Supplementary Figure 8 shows the absorbance versus the wavelength, calculated from the total dielectric function of the bulk material. The absorption spectrum is similar to the UV-Vis-NIR extinction result, but shifted towards higher wavelengths.

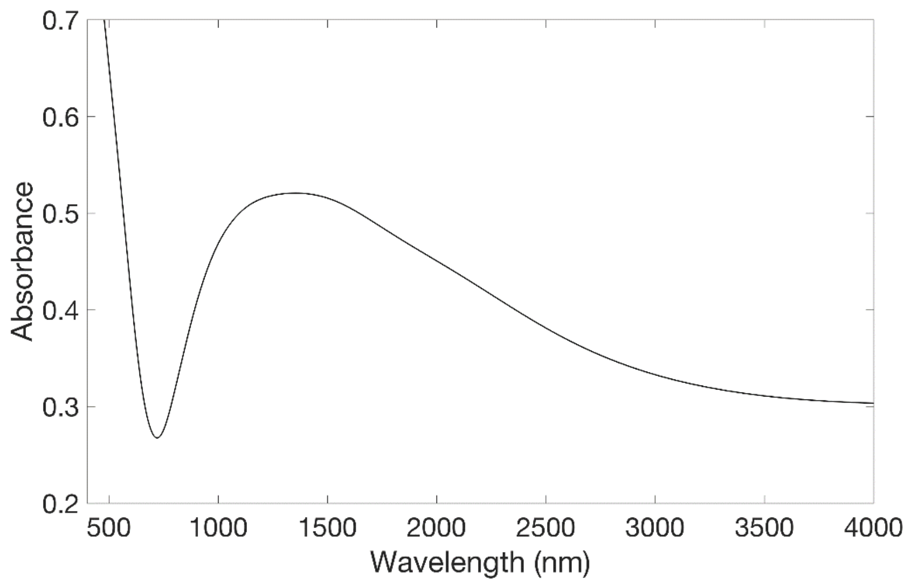


**Supplementary Figure 8.** Absorbance versus wavelength for the bulk material.

In order to include the particle morphology and influence of the surrounding medium, we use the DDSCAT 7.3 code[4], which is based on the discrete dipole approximation (DDA). The absorption and scattering spectra are calculated for a rectangular particle of 25 x 25 x 15 $nm^3$ in a medium with refractive index 1.4968 (toluene) and combined into the extinction spectrum. The result is plotted in Supplementary Figure 9.

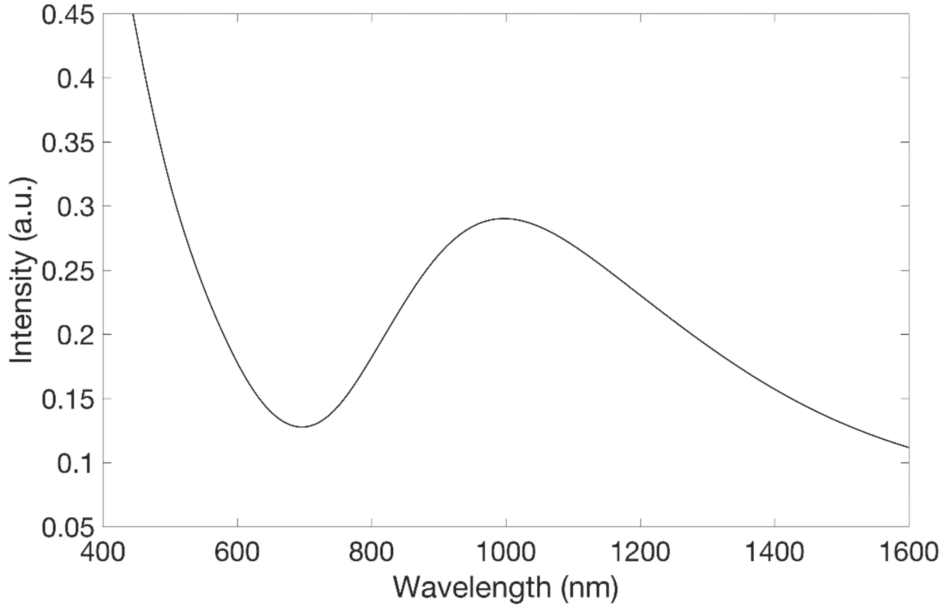


**Supplementary Figure 9.** Extinction spectrum of the 25 x 25 x 15 nm³ rectangular nanoparticle in toluene.

The electron energy loss can be obtained from the energy loss function $L(\omega) = -\mathrm{Im}\left(\varepsilon^{-1}(\omega)\right)$, which is shown in Supplementary Figure 10 for both the unit cell with and without vacancies. The

loss function exhibits a sharp peak with a maximum at 1.40 eV for the proposed unit cell with vacancies.

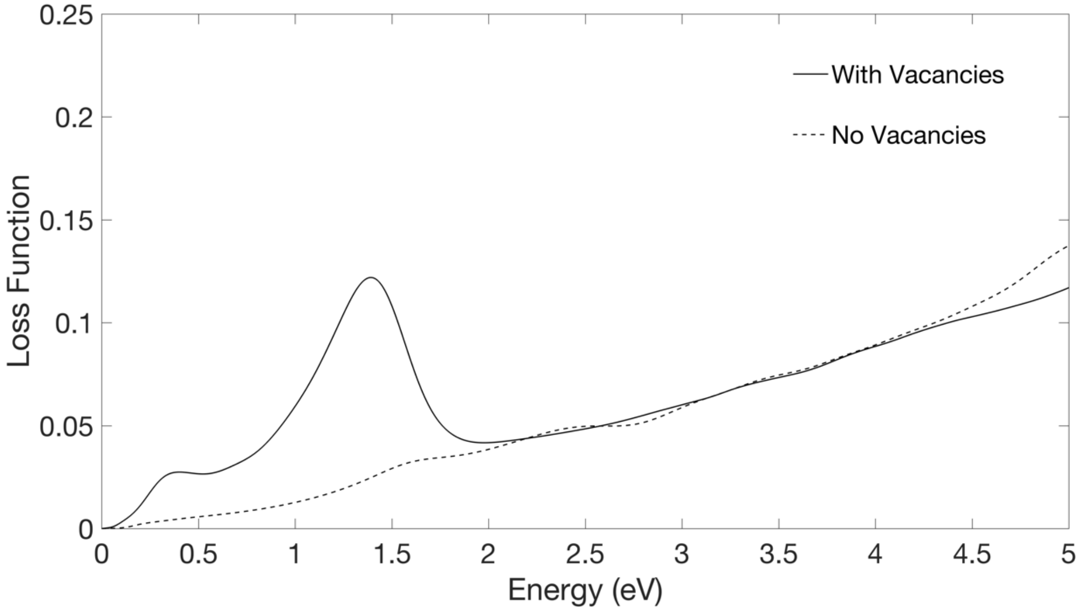


**Supplementary Figure 10.** Loss spectra calculated based on the structure model with all possible Cu sites occupied (dashed line) and the structure model with half of the Cu sites vacant (full line). The structure model without vacancies does not show any loss peak in the energy region 1-2 eV, whereas the structure with vacancies shows a distinct maximum at 1.40 eV.

Supplementary Figure 11 compares the real and imaginary part of the total dielectric function with the calculated loss function over a more extended energy range. The location of the small peak in the low energy range corresponds to the dip in the imaginary part of the dielectric function around 1.4 eV. The broad shoulder around 5 eV corresponds to the zero-crossing of $\varepsilon^{(1)}$ at relatively high values of $\varepsilon^{(2)}$. The large peak around 19 eV in the loss function is produced by the slow zero-crossing of $\varepsilon^{(1)}$ in the energy range where $\varepsilon^{(2)}$ already approaches zero.

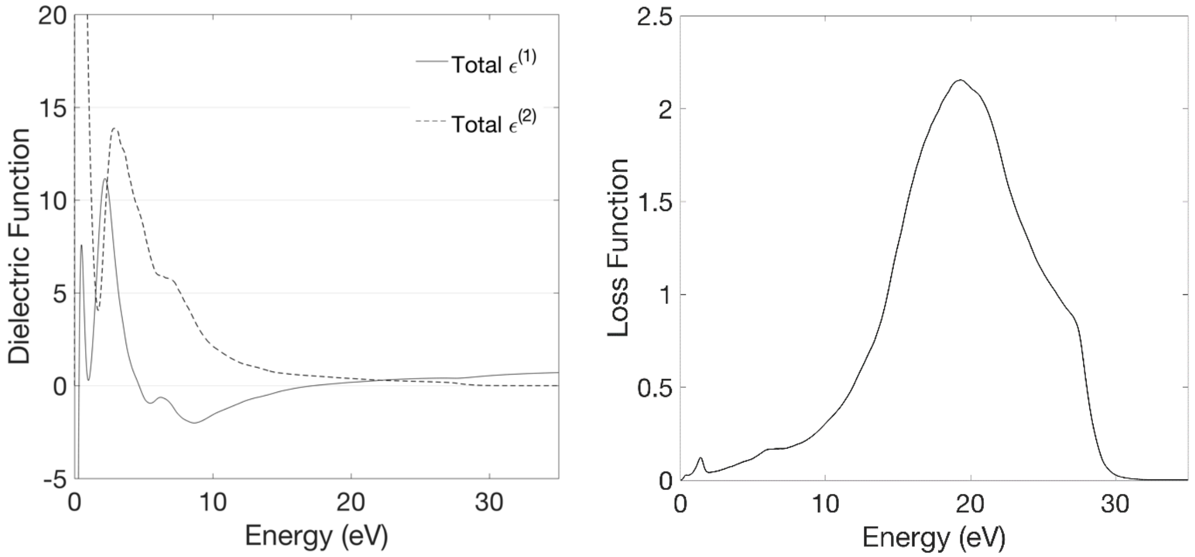


**Supplementary Figure 11.** Real and imaginary part of the dielectric function over a larger energy range (left). The features of the corresponding loss function (right) can be derived from the combination of the real and imaginary part of the dielectric function, as explained in the text.

**Supplementary References**


1. Hohenester, U. & Trügler, A. MNPBEM – A Matlab toolbox for the simulation of plasmonic nanoparticles. *Comput. Phys. Commun.* **183,** 370–381 (2012).
2. Harl, J., Kresse, G., Sun, L. D., Hohage, M. & Zeppenfeld, P. Ab initio reflectance difference spectra of the bare and adsorbate covered Cu(110) surfaces. *Phys. Rev. B* **76,** 035436 (2007).
3. Ambrosch-Draxl, C. & Sofo, J. O. Linear optical properties of solids within the full-potential linearized augmented planewave method. *Comput. Phys. Commun.* **175,** 1–14 (2006).
4. Draine, B. T. & Flatau, P. J. Discrete-Dipole Approximation For Scattering Calculations. *J. Opt. Soc. Am. A* **11,** 1491 (1994).
